# Binding of Thrombin-Activated Platelets to a Fibrin Scaffold through α_IIb_β_3_ Evokes Phosphatidylserine Exposure on Their Cell Surface

**DOI:** 10.1371/journal.pone.0055466

**Published:** 2013-02-01

**Authors:** Tomasz Brzoska, Yuko Suzuki, Hideo Mogami, Hideto Sano, Tetsumei Urano

**Affiliations:** 1 Department of Medical Physiology, Hamamatsu University School of Medicine, Hamamatsu, Japan; 2 Department of Health and Nutritional Sciences, Faculty of Health Promotional Sciences, Hamamatsu University, Hamamatsu, Japan; 3 CREST, Japan Science and Technology Agency, Tokyo, Japan; Heart Center Munich, Germany

## Abstract

Recently, by employing intra-vital confocal microscopy, we demonstrated that platelets expose phosphatidylserine (PS) and fibrin accumulate only in the center of the thrombus but not in its periphery. To address the question how exposure of platelet anionic phospholipids is regulated within the thrombus, an *in-vitro* experiment using diluted platelet-rich plasma was employed, in which the fibrin network was formed in the presence of platelets, and PS exposure on the platelet surface was analyzed using Confocal Laser Scanning Microscopy. Almost all platelets exposed PS after treatment with tissue factor, thrombin or ionomycin. Argatroban abrogated fibrin network formation in all samples, however, platelet PS exposure was inhibited only in tissue factor- and thrombin-treated samples but not in ionomycin-treated samples. FK633, an α_IIb_β_3_ antagonist, and cytochalasin B impaired platelet binding to the fibrin scaffold and significantly reduced PS exposure evoked by thrombin. Gly-Pro-Arg-Pro amide abrogated not only fibrin network formation, but also PS exposure on platelets without suppressing platelet binding to fibrin/fibrinogen. These results suggest that outside-in signals in platelets generated by their binding to the rigid fibrin network are essential for PS exposure after thrombin treatment.

## Introduction

Adequate exposure of anionic phospholipids on platelet surfaces, which is required for the promotion and regulation of coagulation, is essential for normal hemostasis [1], [2]. Non-activated platelets keep a dynamic asymmetric steady-state of their membranes in which procoagulant phosphatidylserine (PS) is held in the inner leaflet. Upon stimulation with an agonist, morphological changes as well as transient elevation of the intracellular calcium concentration ([Ca^2+^]_i_) take place in platelets. Subsequent events trigger additional secretion of Ca^2+^-mobilizing agonists, such as adenosine diphosphate (ADP), from dense granules and cause Ca^2+^ influx, which in turn results in sustained elevation of [Ca^2+^]_i_ in platelets. This activates Ca^2+^-dependent scramblases such as transmembrane protein 16F (TMEM16F) [3] and inhibits translocase. Ultimately anionic phospholipids, among which PS is the most effective, can be relocated within the platelet membrane. The exposure of PS on activated platelets promotes thrombin formation by providing a catalytic membrane surface for tenase and prothrombinase assembly and activity [1], [2], [4]. The physiological importance of PS exposure for normal hemostasis is demonstrated by Scott syndrome, which is a rare congenital bleeding disorder that originates from a defective scramblase mechanism [1]. Employing intravital confocal microscopy, we recently demonstrated that platelets can be fully activated and expose PS on their cell surface only when they exist in the center of the thrombus but not when they are located in its periphery. The localization of PS-exposing platelets in the core of the intravascular thrombus was similar to that of fibrin [4]. These results suggest that the exposure of platelet anionic phospholipids is precisely regulated by time- and space-dependent regulatory mechanisms, which are essential to quickly produce enough amounts of thrombus to stop bleeding as well as to prevent the generation of thrombus in excess. The exact mechanism and involved factors that regulate PS exposure during thrombus formation, however, remain to be defined. To evaluate how PS exposure is modulated in platelets within a thrombus, an *in-vitro* experiment was employed in which a fibrin network was formed in the presence of platelets, and PS exposure on the platelet surface was analyzed by confocal laser scanning microscopy. Alteration of fibrin network formation by several different methods suppressed platelet PS exposure, suggesting that crosstalk between platelets and the fibrin scaffold is a key feature of the exposure of anionic phospholipids.

## Materials and Methods

### Ethics Statement

Experimental protocol was approved by the Hamamatsu University School of Medicine ethics committee and all blood donors provided written informed consent.

### Reagents

The following materials were purchased from the indicated sources: rhodamine-6G (R-6G) (Tokyo Chemical Industry Co., Ltd., Tokyo, Japan), Alexa Fluor 488 and 647, fluo-4 AM and Fura Red AM (Molecular Probes, Eugene, OR, USA), ADP and collagen (Siemens Healthcare Diagnostics, Marburg, Germany), recombinant tissue factor (TF)(Instrumentation Laboratory, Lexington, MA, USA). Ionomycin (IMC), thrombin, cytochalasin B (Cyt-B) and Gly-Pro-Arg-Pro amide (GPRP) were purchased from Sigma (St. Louis, MO, USA). Cytochalasin B was dissolved in DMSO and GPRP in 0.9% NaCl. Annexin A5 (annexin V) was donated by KOWA Pharmaceuticals (Tokyo, Japan). Human fibrinogen was purchased from Enzyme Research Laboratories (South Bend, IN, USA). Argatroban was obtained from Mitsubishi Pharma Corporation (Osaka, Japan) and dissolved in 0.9% NaCl containing 1.0 M equivalent of HCl, FK633 was provided by Astellas Pharma Inc. (Tokyo, Japan) and dissolved in DMSO. Gly-Pro-Pro-Pro (GPPP) was purchased from Funakoshi Co. (Tokyo, Japan) and dissolved in 0.9% NaCl. Arg-Gly-Asp-Ser (RGDS) and Arg-Gly-Glu-Ser (RGES) tetrapeptides were purchased from Abbiotec (San Diego, CA, USA) and dissolved in 0.9% NaCl.

### Platelet preparation

Blood samples from healthy adult donors were collected in 0.1 volumes of 3.8% trisodium citrate. Platelet-poor plasma (PPP) was prepared by centrifugation at 1,800 *g* for 10 minutes at 22°C. To stain platelets, whole blood was incubated with R-6G (0.05%, 5 µl/ml whole blood) for 15 minutes in the dark at room temperature, and subsequently centrifuged at 250 *g* for 10 minutes at 22°C to obtain platelet-rich plasma (PRP). PRP containing non-labeled platelets was also prepared as needed. Employing of a whole-blood cell counter (Celltac α, Nihon Kohden, Tokyo, Japan) we ensured the purity of PRP and determined the platelet concentration.

For the preparation of suspension of washed platelets, the method developed by Mustard et al. [5] was used with some modifications. PRP containing labeled with R-6G platelets was centrifuged at 1,100 *g* for 15 min at 22°C. PPP was removed and the platelets were resuspended in Tyrode's albumin buffer (NaCl 137 mM, KCl 2.7 mM, NaHCO_3_ 10 mM, NaH_2_PO_4_ 0.36 mM, CaCl_2_ 1 mM, MgCl_2_ 1 mM, HEPES 5 mM, glucose 1 g/L, bovine serum albumin 3.5 g/L, pH 7.35) containing 0.5 µM of prostacyclin and centrifuged at 950 *g* for 10 min at 22°C. Subsequently washing step was repeated and the platelet pellet was resuspended in Tyrode's albumin buffer containing no prostacyclin eventually. Platelet count was determined using a whole-blood cell counter. Before each experiment platelets were studied microscopically to ensure the absence of platelet aggregates and minimal presence of contaminating cells.

All platelet samples were used within 6 hours after blood collection.

### Platelet aggregation studies in vitro

Platelet aggregation was measured in samples containing PRP mixed with PPP (final volume 150 µl, 2.5×10^5^ platelets/µl) and 50 µl of 0.9% NaCl by the turbidimetric method of Born [6] employing a lumiaggregometer (PAT-2M, SSR Engineering Co., Ltd., Tokyo, Japan). The aggregometer was calibrated with a sample containing platelets for zero light transmission and a sample containing 150 µl of PPP and 50 µl of 0.9% NaCl for 100% transmission. Samples were stirred at 1,000 rpm. Platelet aggregation agonists (ADP, 4.5 µM or collagen, 5 µg/ml or 0.18 mg/ml) were added, and the changes in relative light transmittance produced an aggregation curve over 10 minutes at 37°C. The inhibition of aggregation in drug-treated samples was expressed as a percentage of the aggregation in the control sample. In experiments with Cyt-B, platelets were pre-incubated with Cyt-B for 10 minutes before stimulation by each agonist.

### Clot retraction assay

Clot retraction was quantified by measuring the time-dependent decrease in the surface area of plasma clots formed in 35-mm glass bottom dishes at 37°C in the presence of platelets (1.0×10^8^ cells/ml). Clotting reactions were initiated by addition of 1 U/ml thrombin and 10 mM CaCl_2_ to 160 µl of PRP (1.25×10^8^ cells/ml). The total volume of each sample was 200 µl. In experiments with Cyt-B (100 µg/ml) PRP was pre-incubated with the drug for 10 minutes before the initiation of clot formation. FK633 (30 µM) was mixed with PRP just before the initiation of clot formation. The plasma clot surface area was calculated by Adobe Photoshop CS5 from a digital image taken 60 minutes after clot formation. The percent inhibition was calculated by considering clot retraction in the absence of pharmacological reagents to be a full retraction.

### Confocal laser scanning microscopy

A confocal laser scanning microscope (CLSM; FV1000, Olympus) equipped with a 100× (NA 1.40) oil immersion objective lens and a temperature control system was used. All experiments were conducted in 35-mm glass bottom dishes at 37°C.

Platelet suspensions in plasma(100 µl, 2.0×10^4^ platelets/µl) were obtained by supplementation of PPP with PRP containing platelets labeled with R-6G. Subsequently, the suspension was adequately replenished with either 0.9% NaCl, annexin V (22.5 nM) labeled with Alexa Fluor 647 (ANX) and human fibrinogen (15 µg/ml) labeled with Alexa Fluor 488 (fbg-488) when platelet surface PS exposure was calculated [4], [7], [8], [9], [10], or with human fibrinogen (15 µg/ml) labeled with Alexa Fluor 647 (fbg-647) when binding of fibrinogen to platelets was evaluated. Annexin V and fibrinogen were labeled with the fluorescent dyes according to the manufacturer protocols. The desired pharmacological reagent was simultaneously supplemented as needed, and the experiment commenced when CaCl_2_ (10 mM) and a chosen compound (TF, IMC, thrombin) were added to initiate clot formation. The total volume of each sample was 200 µl. In experiments with Cyt-B platelets were pre-incubated with Cyt-B for 10 minutes at 37°C before being stimulated with either thrombin or IMC.

The suspension of washed platelets in Tyrode's albumin buffer was supplemented with ANX (22.5 nM) and GPRP (3 mM) if needed. As previously, the experiment began when CaCl_2_ (10 mM) and thrombin (1 U/ml) were added. The final concentration of platelets in each 200 µl sample was 1.0×10^4^ platelets/µl. Immediately after agonist addition, the focal plane approximately 3 µm above the bottom of the dish, in a randomly selected location, was chosen and images were taken every 20 or 35 s. Subsequent to fibrin network formation, a constant observation field was kept for the entire duration of the experiment. When a fibrin clot was not formed a z-stack of 10 optical sections, collected at 1.11 µm intervals, at up to 1 frame per 20 s from the bottom to the interior of the dish, was captured every 5 minutes. The calculated optical thickness of each slice was 0.93 µm. Collected images were analyzed using FV10-ASW software (Olympus), and data were expressed as the percentage of fluorescence-positive platelets.

### Concurrent monitoring of platelet intracellular Ca^2+^ concentration changes and PS exposure

PRP was prepared as described above and concentration of platelets was adjusted to 3×10^5^ cells per microliter with autologous PPP. For measurement of platelet [Ca^2+^]_i_ PRP was incubated with 1 µM fluo-4 AM together with 10 µM Fura Red AM for precisely 45 min at room temperature. Subsequently platelet suspension (100 µl, 2.0×10^4^ platelets/µl) was obtained by supplementation of PPP with PRP containing platelets loaded with fluorescent dye. Next, the suspension was adequately replenished with either 0.9% NaCl and ANX (22.5 nM). The experiment commenced when CaCl_2_ (10 mM) and thrombin (1 U/ml) were added to initiate clot formation. The total volume of each sample was 200 µl. Studies were conducted in 35-mm glass bottom dishes at 37°C using CLSM as described above, though images were taken every 4.4 s. Collected images were analyzed using FV10-ASW (Olympus) and AquaCosmos 2.6 (Hamamatsu Photonics, Hamamatsu, Japan) software. The fluorescence intensity was normalized to each maximal value; the relative fluorescence change is referred to as the “relative fluorescence intensity”. The fluo-4 to Fura Red relative fluorescence intensities ratios were calculated and then used as an index of the relative change in [Ca^2+^]_i_.

### Statistical analysis

Results are reported as means ± standard deviations (SD). Statistical comparisons of samples were conducted using the Student's t-test. Differences were considered significant at P<0.05.

## Results

### Validation of the ability of R-6G labeled platelets to aggregate in response to collagen and ADP using a turbidimetric aggregometer

To characterize the platelet population used in our studies, abilities of non-labeled platelets and platelets labeled with R-6G to aggregate in response to either collagen or ADP stimulation were compared. No significant differences were revealed between these samples in three separate experiments. The respective suspensions showed comparable responses to both stimuli (Figure S1).

### TF and thrombin effects on fibrin network formation and platelet PS exposure in CLSM studies

**Figure 1:**
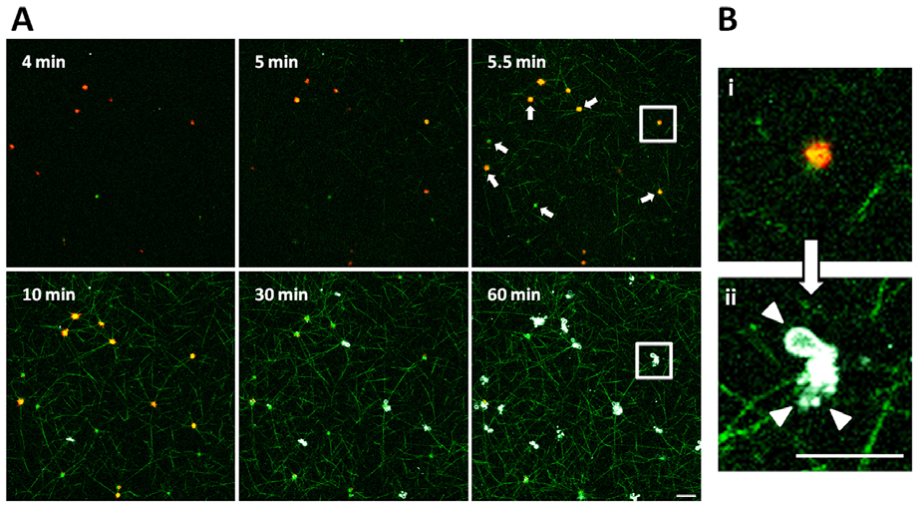
TF supplementation evokes fibrin network formation as well as PS exposure. [41] CLSM images show fibrin network (green) formation in the presence of platelets labeled with R-6G (red). TF addition evoked fibrin matrix assembly and concurrent immediate binding of the fibrin/fibrinogen by platelets (white arrows) followed by progressive PS exposure on the platelet surface which was detected by platelet ANX (white) binding. (Bi) An enlarged representative image of single platelet bound to fibrin scaffold before the anionic phospholipids exposure in the outer leaflet of membrane, and (Bii) morphological changes of platelet membrane such as blebbing and shedding of microvesicles from cell surface (arrow heads) which accompany PS exposure. Scale bars show 10 µm.

**Figure 2:**
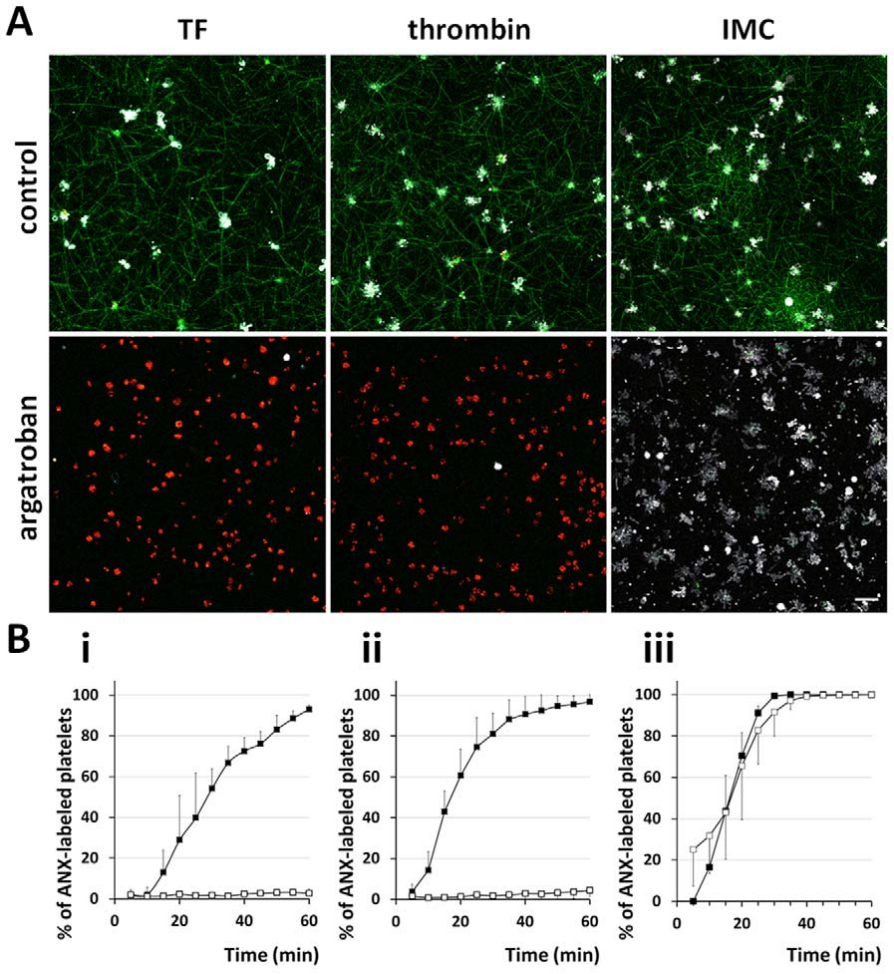
Effect of a direct thrombin inhibitor on platelet PS exposure. (A) CLSM images obtained at the level of 3 µm from the bottom of the dish 60 minutes after supplementation of diluted PRP, containing R-6G labeled platelets (red), fbg-488 (green), ANX (white), with either TF, thrombin (1 U/ml) or IMC (10 µM) in the absence and presence of argatroban (100 µM), respectively. In argatroban-treated samples fibrin network formation was abrogated, therefore with time course we could observe falling of platelets to the bottom of the dish, thus ultimately the number of platelets at the bottom of the dish in non treated and treated with argatroban samples is different. (B) Kinetics of platelet PS exposure expressed as the percentage of ANX fluorescence-positive platelets in CLSM studies. Graphs present exposure of platelet PS in absence (closed square) and presence of 100 µM argatroban (open square) upon supplementation of either (i) TF (n = 5 and n = 6, respectively), (ii) thrombin (1 U/ml, n = 5 and n = 7, respectively) or (iii) IMC (10 µM, n = 5 and n = 7, respectively). Data are shown as mean ± SD. Scale bar shows 10 µm.

CLSM imaging was employed to analyze platelet PS exposure, and thus platelet procoagulant activity, in a fibrin clot. In order to resemble the physiology of hemostasis at the outset TF was used to initiate the coagulation cascade, imitating thrombin generation *in-vivo* and consequent fibrin formation [11], [12]. Sequentially diluted concentrations of TF were used to initiate fibrin clot formation in this system, and finally 8000 times diluted recombinant TF (approximately 1/16000 of the concentration used to assay prothrombin time) was employed to enable clot formation after approximately 5 minutes. After TF addition, platelets initially drifted freely in the samples. Fibrin networks were formed subsequently, and ultimately all platelets invariably bound fibrin and became incorporated into the fibrin mesh. In some cases fibrin assembly was preceded by PS exposure by a single platelet. Formation of a fibrin scaffold was invariably followed by progressive full activation of platelets, as indicated by ANX binding to platelet surfaces that exposed PS [4], [7], [8], [9], [10] and by R-6G loss possibly due to the change of platelet membrane permeability or by R-6G translocation evoked by the alteration of platelet membrane potentials (Figure 1) [4], [13]. Loss of membrane lipid asymmetry was often accompanied by blebbing and subsequent shedding of lipid-symmetric microvesicles from the cell surfaces (Figure 1B). On average, the fibrin network started to form at 5.7±1.8 minutes (mean ± SD, n = 5) after TF addition and 93.1±2.0% (mean ± SD, n = 5) of cells exposed PS within 60 minutes of the experiment. Argatroban, a direct thrombin inhibitor [12], [14], at a concentration of 100 µM restrained not only fibrin assembly but also PS exposure, and only 2.8±1.9% (mean ± SD, n = 6) of these platelets exposed PS one hour after TF supplementation (Figure 2A,Bi).

Thrombin-evoked fibrin network formation and full activation of platelets proceeded in an analogous way to TF-evoked processes. Thrombin at a concentration of 1 U/ml induced fibrin scaffold formation within 3.8±2.4 minutes (mean ± SD, n = 5). Following agonist addition, fibrin mesh assembly and fibrin binding, platelets started to increasingly expose PS and 96.8±3.6% (mean ± SD, n = 5) of the cells became fully activated after 60 minutes. Conversely, argatroban at a concentration of 100 µM again entirely inhibited fibrin assembly, and 60 minutes after thrombin addition only a very small number of platelets bound ANX (4.4±3.1%, mean ± SD, n = 7) (Figure 2A,Bii). These results confirm that although TF was employed to mimic the physiological process of extrinsic coagulation pathway, thrombin was the essential molecule that not only directly induced fibrin network formation but also evoked exposure of anionic phospholipids in the outer leaflet of platelet membranes.

**Figure 3:**
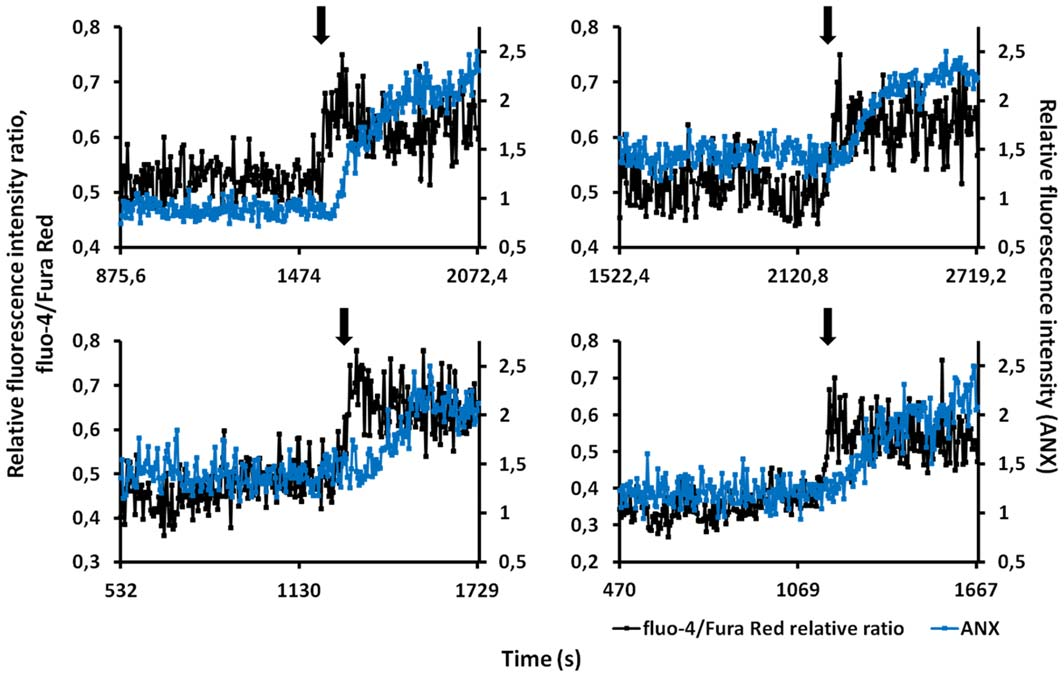
[Ca^2+^]_i_ elevation and PS exposure in platelets stimulated by thrombin and bound to fibrin network. Representative traces show [Ca^2+^]_i_ changes illustrated as fluo-4 to Fura Red relative fluorescence intensities ratios (black) in four randomly chosen platelets bound to rigid network of fibrin (n = 37 cells from four independent experiments). There were essentially no changes in the fluorescence intensities of ANX (blue) while spontaneous Ca^2+^ oscillations were observed in platelets. Ultimately, platelet morphological alterations and PS exposures were directly preceded by the sustained [Ca^2+^]_i_ elevations (arrows).

Since platelet anionic phospholipids exposure depends strictly on the [Ca^2+^]_i_ elevation, we tried to measure [Ca^2+^]_i_ in platelets in order to evaluate its relationship with PS exposure in our studies employing two different kinds of [Ca^2+^]_i_ indicators. The use of dual indicators to study intracellular calcium dynamics minimizes the contribution of artifactual changes in the fluorescence signal not related to changes in [Ca^2+^]_i_, e.g., the slight fibrin network movement and nonuniform dye loading [15]. We applied this principle here to study [Ca^2+^]_i_ dynamics in thrombin treated and bound to the fibrin matrix platelets using the combination of fluo-4 AM and Fura Red AM. In this manner, an increase in [Ca^2+^]_i_ was monitored by an increase in the fluorescence of fluo-4 as well as by a simultaneous reduction of that of Fura Red. We then calculated the emission ratio of fluo-4 to Fura Red as a ratiometric indicator which increases according to [Ca^2+^]_i_ elevation and is independent of the changes of local dye concentration that occur during platelet morphological rearrangements [16]. We also concurrently monitored changes in relative fluorescence intensity of ANX in platelets bound to the fibrin matrix. Thrombin (1 U/ml) evoked fibrin network formation within 3.4±1.1 minutes (mean ± SD, n = 4). Bound to the fibrin matrix platelets initially showed spontaneous Ca^2+^ oscillations with negligible changes in ANX fluorescence intensity. Ultimately, ANX fluorescence intensity increased, which was always preceded by the increase in relative fluorescence intensity ratio of fluo-4/Fura Red (Figure 3). Thus sustained elevation of [Ca^2+^]_i_ was clearly proved in platelets which exposed PS on their surface after treatment by thrombin and binding to the fibrin matrix.

### [Ca^2+^]_i_ - dependent platelet PS exposure upon IMC stimulation

Since our results have demonstrated evident interdependence between the [Ca^2+^]_i_ and the platelet PS exposure we used 10 µM IMC, a Ca^2+^ ionophore [17], to evaluate its effect in CLSM studies. Fibrin networks were formed 6.1±2.7 minutes (mean ± SD, n = 5) after ionophore addition. Again we observed spreading of the fibrin network and concurrent binding of all platelets to fibrin, however, the kinetics of PS exposure seemed to be faster than those in previous experiments, most likely as a result of the [Ca^2+^]_i_ increase evoked by IMC. In the present study 99.4±1.3% (mean ± SD, n = 5) and all of the platelets were activated after 30 and 60 minutes, respectively (Figure 2A,Biii). In argatroban-treated samples the fibrin network was not formed (Figure 2A), nonetheless, during the experiment we observed the successive binding of ANX to platelets. Within the first 30 minutes of the experiment 91.6±11.7% (mean ± SD, n = 7) of the cells became fully activated, and all were activated at the experiment's end. The differences in PS exposure between samples with and without supplemented argatroban were not significant (Figure 2Biii). Taken together, these results confirm that sustained elevation of [Ca^2+^]_i_ is crucial for PS exposure even in the absence of the fibrin matrix. Thus the generation of platelet procoagulant activity involves Ca^2+^-dependent activation of scramblase and Ca^2+^-dependent inhibition of translocase [2], [4], [18].

### α_IIb_β_3_ role in promotion of platelet procoagulant activity

Platelet integrin α_IIb_β_3_ is essential for and directly involved in adhesion, aggregation, clot retraction and spreading of platelets upon activation [19], [20]. Therefore, FK633, a selective reversible antagonist of α_IIb_β_3_ [21] was used at a dose of 30 µM to clarify the role of α_IIb_β_3_ in platelet PS exposure. FK633 almost completely restrained platelet aggregation induced by collagen (0.18 mg/ml) or ADP by 95.1±3.0% (mean ± SD, n = 3) and 98.6±2.5% (mean ± SD, n = 3) respectively at a concentration of 30 µM. In three independent trials, FK633 entirely abrogated clot retraction at this concentration (data not shown). DMSO, which was used to dissolve FK633, was confirmed to show only an insignificant effect on platelet aggregation and PS exposure on the platelet surface in CLSM experiments as well as clot retraction at an employed concentration of 0.5% (data not shown).

**Figure 4:**
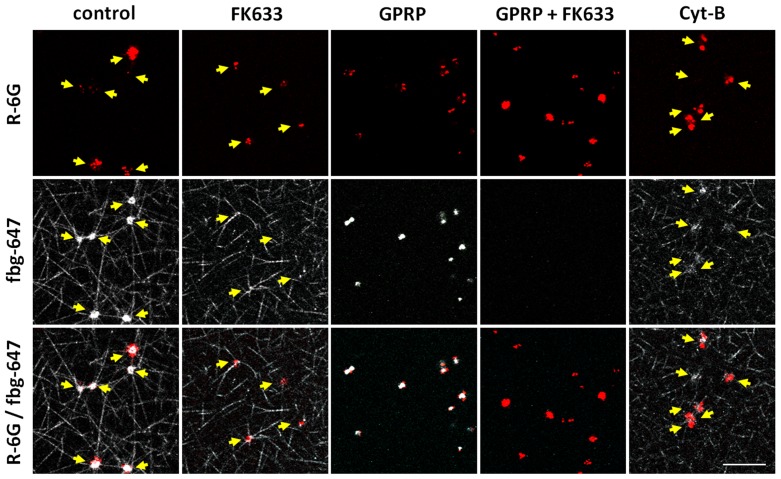
Fibrin scaffold structure and platelet fibrin/fibrinogen binding in CLSM studies. CLSM images were taken 15 minutes after stimulation of diluted PRP with thrombin (1 U/ml) in order to analyze fibrin network formation in the presence of platelets. Fluorescence images of R-6G (red), which was loaded to platelets, are shown in the upper panel. Fluorescence images of fibrinogen labeled with Alexa Fluor 647 (white) are shown in the middle panel as either fibrin network or fibrin/fibrinogen binding to platelets surface. The merged figures are shown at the bottom panel. In the control essentially all the platelets were bound to fibrin network (white) and those surfaces were coated with either fibrin or fibrinogen (white), though some of them already released rhodamine-6G as a result of substantial activation. In the presence of FK633 (30 µM), though fibrin network formation was observed, platelets were neither bound to fibrin network nor coated with fibrin/fibrinogen. In contrary, in the presence of GPRP (3 mM), fibrin network was not formed but platelets were coated with fibrin/fibrinogen. In the presence of both FK633 and GPRP, neither fibrin network formation nor the binding of fibrin/fibrinogen to platelets were observed. Ultimately, in the presence of Cyt-B (100 µg/ml) fibrin mesh formation was not disturbed, however platelets were coated with fibrin/fibrinogen in a restricted manner only. Arrows indicate localizations of platelets within the fibrin mesh. Scale bar shows 10 µm.

**Figure 5:**
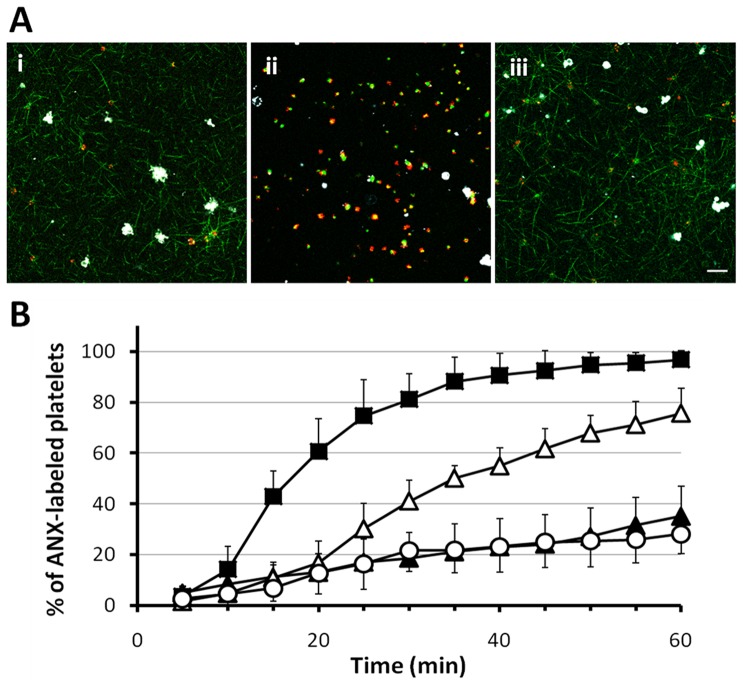
Attenuation of fibrin network formation suppress PS exposure on platelets surface. (A) Exposure of PS by R-6G labeled platelets (red) in the presence of either (i) FK633 (30 µM), (ii) GPRP (3 mM) or (iii) Cyt-B (100 µg/ml) upon stimulation of diluted PRP containing fbg-488 (green) and ANX (white) with thrombin (1 U/ml). CLSM images were taken 60 minutes after thrombin supplementation. (B) Kinetics of platelet PS exposure expressed as the percentage of ANX fluorescence-positive platelets in thrombin-treated (1 U/ml) samples in CLSM study. Presence of either FK633 (open triangle, n = 7), GPRP (open circle, n = 7) or Cyt-B (closed triangle, n = 7) suppressed platelet anionic phospholipids exposure compared with control (close square, n = 5), suggesting that crosstalk between platelets and the fibrin scaffold is a key feature of the anionic phospholipids exposure. Data are shown as mean ± SD. Scale bar shows 10 µm.

Since earlier CLSM studies showed that there were no significant differences in platelet PS exposure and fibrin network formation upon either TF or thrombin supplementation, we decided to use 1 U/ml of thrombin as an agonist to mimic TF-evoked fibrin assembly and platelet PS exposure in further studies evaluating the effects of several pharmacological compounds. In the presence of FK633, thrombin initiated the formation of a fibrin network at 3.8±2.0 minutes (mean ± SD, n = 7), which is equivalent to results obtained in the absence of FK633. Despite the thrombin-induced fibrin mesh formation, platelet binding of fibrin/fibrinogen was mostly inhibited in the presence of FK633 (Figure 4), however, we still could observe some platelets that were spreading and extruding pseudopods. Notwithstanding the slower kinetics of ANX binding, especially in the initial phase of the experiment, the final platelet PS exposure was moderately but still significantly reduced to 75.7±9.9% (mean ± SD, n = 7) after 60 minutes (Figure 5Ai,B).

Subsequently, IMC, a direct stimulator to increase [Ca^2+^]_i_, was used to exclude the possibility that FK633 interfered the machinery of PS exposure. In the presence of FK633 93±7.7% (mean ± SD, n = 5) and 100% of the IMC-stimulated platelets bound ANX within 30 and 60 minutes of the experiment, respectively.

RGDS tetrapeptide is well known inhibitor of α_IIb_β_3_ receptor fibrinogen binding [22]. In our studies RGDS at a concentration of 1.2 mM fully suppressed platelet aggregation induced by collagen (0.18 mg/ml) or ADP (Figure S2A,B). In the presence of the control peptide, RGES (1.2 mM), ADP-induced platelet aggregation was not interfered, however, collagen-evoked platelet aggregation was significantly delayed (Figure S2A,B). In CLSM studies fibrin network formation was not disrupted either in the presence of RGDS or RGES, and started 1.9±0.6 minutes (mean ± SD, n = 5) and 2.3±0.7 minutes (mean ± SD, n = 3) after thrombin addition, respectively. The RGDS supplementation significantly reduced the kinetics of platelet PS exposure, even so 92.4±2.3% (mean ± SD, n = 5) of platelets bound ANX at the end of experiment (Figure S2C). RGES tetrapeptide retarded the platelet PS exposure kinetics in a less extended manner and ultimately 98.2±1.7% (mean ± SD, n = 3) of the platelets bound ANX within 60 minutes of experiment (Figure S2C).

Together, these results suggest that α_IIb_β_3_ occupancy plays an important role in the modulation of platelet procoagulant activity.

### GPRP inhibits fibrin network formation and platelet PS exposure

GPRP is a synthetic peptide that inhibits fibrin polymerization and thus thrombin induced plasma coagulation by binding to the carboxy-terminal region of the γ-chain located in the D domain of fibrin [23], [24]. GPRP also inhibits ADP-evoked platelet aggregation by inhibiting fibrinogen binding to α_IIb_β_3_ [25], [26]. Indeed, GPRP at a concentration of 3 mM reduced ADP-induced aggregation of platelets by 77.8±5.5% (mean ± SD, n = 4). Subsequently, we performed a series of experiments employing a CLSM. We first evaluated fibrin/fibrinogen binding to platelets in the presence of 3 mM GPRP. Although fibrin network formation was successfully inhibited by GPRP, we clearly observed rapid progressive binding of fibrin/fibrinogen by platelets (92.5±10.8, 97.9±2.4 and 98.2±1.5% at 5, 30 and 60 minutes after thrombin stimulation, mean ± SD, n = 7). Some platelets appeared to stick together via fibrin/fibrinogen and created small aggregates. A combination of 3 mM of GPRP together with 30 µM of FK633 entirely inhibited platelet fibrin/fibrinogen binding in three separate experiments (Figure 4), suggesting that the binding of platelets to fibrin/fibrinogen in the presence of GPRP alone was α_IIb_β_3_ dependent. Next, exposure of platelet PS upon 1 U/ml thrombin stimulation in the presence of GPRP was evaluated. Binding of ANX to platelets was greatly diminished and reached only 30.7±4.0% (mean ± SD, n = 7) within 60 minutes after thrombin addition (Figure 5Aii,B). In contrary, in the presence of GPPP, a control peptide, thrombin initiated the assembly of a fibrin mesh at 2.8±1.3 minutes (mean ± SD, n = 4). Subsequently, platelets exposed PS in a time dependent manner and 96.6±4.0% (mean ± SD, n = 4) of platelets bound ANX at 60 minutes.

As expected, in IMC-treated PRP GPRP restrained fibrin network formation, but not PS exposure. Finally 92.6±11.6% (mean ± SD, n = 4) and all of platelets bound ANX after 30 and 60 minutes, respectively.

Ultimately, washed platelets were used to exclude the possibility of suppression of platelet anionic phospholipids exposure by GPRP independently of fibrin. PS exposure on thrombin stimulated washed platelets in the presence of GPRP (39.3±7.8, mean ± SD, n = 4) was similar to that in the absence of GPRP (41.5±9.1%, mean ± SD, n = 5), implying that the inhibitory effect of GPRP was fibrin/fibrinogen dependent.

Taken together, these data strongly suggest that, thrombin-evoked surface exposure of platelet anionic phospholipids requires the presence of a fibrin scaffold.

### Role of cytoskeleton in platelet exposure of PS

The cytoskeleton actively participates in the platelet hemostatic response [27]. To determine involvement of the cytoskeleton in the regulation of platelet procoagulant activity, Cyt-B at a final concentration of 100 µg/ml was used to inhibit actin polymerization, and thus cytoskeleton reorganization, upon direct platelet activation by 1 U/ml of thrombin [27] DMSO, which was used to dissolve Cyt-B, was confirmed to show only a negligible effect on both platelet aggregation and PS exposure on the platelet surface in CLSM experiments at a concentration of 0.8% (data not shown).

Ultimately, Cyt-B profoundly inhibited platelet aggregation evoked by collagen (0.18 mg/ml) by 94.4±0.9% (mean ± SD, n = 3) or ADP by 74.5±6.9% (mean ± SD, n = 3). Clot retraction was entirely abrogated in three independent experiments (data not shown). Although in CLSM studies fibrin network formation was not disrupted and started 2.1±0.8 minutes (mean ± SD, n = 7) after thrombin addition, Cyt-B markedly limited fibrin/fibrinogen binding by platelets (Figure 4). Moreover, upon inhibition of actin polymerization, platelets maintained their rounded, regular shape even after fibrin assembly. Cyt-B strongly suppressed PS exposure, and only 35.1±11.8% (mean ± SD, n = 7) of platelets bound ANX at 60 minutes (Figure 5Aiii,B).

**Figure 6:**
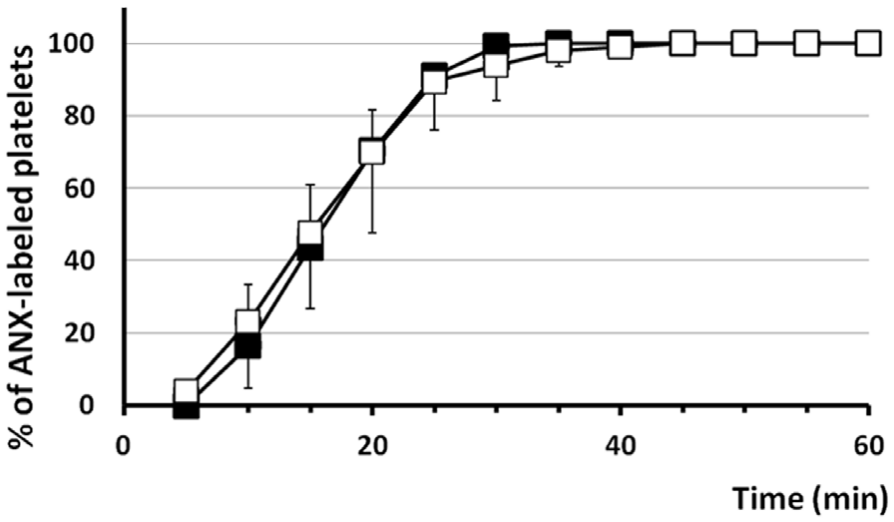
Cyt-B does not affect IMC dependent platelet anionic phospholipids exposure. Kinetics of platelet PS exposure are expressed as the percentage of ANX fluorescence-positive platelets in CLSM studies. Graph presents exposure of platelet PS either in the absence (closed square, n = 5) or the presence of 100 µg/ml Cyt-B (open square, n = 6) upon supplementation of IMC at a concentration of 10 µM. Data are shown as mean ± SD.

Subsequently IMC (10 µM) was used in CLSM experiments to address the question whether Cyt-B (100 µg/ml) affects ionophore dependent platelet PS exposure. In the presence of the actin polymerization inhibitor, IMC initiated the formation of a fibrin network at 8.0±1.4 minutes (mean ± SD, n = 6). In the present study 93.9±9.8% (mean ± SD, n = 6) and all of the platelets were activated after 30 and 60 minutes, respectively (Figure 6). Together, these results indicated that the proper cytoskeleton activity is indispensable for platelets to efficiently expose PS. Moreover, Cyt-B does not interfere with the machinery of PS exposure that is induced by direct stimuli to increase [Ca^2+^]_i_.

## Discussion

Based on our recent findings that PS-exposing platelets were unevenly distributed and mostly localized at the core of the micro thrombus [4], we presumed that mechanical foci, which differ significantly and depend on the localization in micro thrombus, could be one of the factors that regulate PS exposure in platelets. In the present study, by employing CLSM we clarified that mechanical foci, generated by the fibrin network formed around platelets and transmitted to platelets through α_IIb_β_3_, are essential for PS exposure in TF-dependent plasma clots.

It is well known that sustained elevation of [Ca^2+^]_i_ is necessary for anionic phospholipids exposure, and thus calcium ionophore triggers PS exposure as also shown in the present study (Figure 2Biii). Nonetheless, little is known about the physiological stimuli that evoke PS exposure. Only a limited fraction of platelets (10–20%) exposes PS upon adhesion to collagen together with thrombin stimulation [28]. In present study we employed diluted PRP that was treated with either TF or thrombin, and subsequently PS exposure in platelets surrounded by the generated fibrin network was analyzed. After treatment by either TF or thrombin, most platelets expressed PS on their surface, similar to earlier observations of platelets in the center of thrombus in an *in-vivo* system. Both agents had much the same effect on fibrin network formation, and insignificant differences in exposure of platelet PS were detected after 60 minutes. The presence of argatroban, a direct thrombin inhibitor, restrained both fibrin assembly and PS exposure induced not only by thrombin but also by TF supplementation (Figure 2A,Bi,ii). In the case of IMC, which causes [Ca^2+^]_i_ elevation followed by inhibition of the translocase and activation of the scramblase, the PS exposure was not abrogated by argatroban (Figure 2A,Biii), but fibrin assembly was inhibited. In contrast, vehicles did not exert any impact on TF-, thrombin- or IMC-induced fibrin mesh formation as well as platelet PS exposure in CLSM studies (data not shown). Taken together, these results suggest that exposure of platelet anionic phospholipids triggered by TF is primarily induced by generated thrombin, and moreover argatroban does not interfere with the machinery of PS exposure that is induced by direct stimuli to increase [Ca^2+^]_i_. Based on these findings we decided to use thrombin to further analyze the mechanism by which platelets incorporated into the fibrin network express PS.

Since platelets bind both fibrin and fibrinogen through α_IIb_β_3_, we first analyzed the effect of an α_IIb_β_3_ antagonist on PS exposure. The inhibitory effect of FK633 on clot retraction and platelet aggregation confirmed the fundamental characteristics of this drug and the appropriateness of its usage in this investigation. CLSM studies showed clear abrogation of fibrin/fibrinogen binding by platelets in the presence of FK633 (Figure 4). Platelet shape changes and pseudopods protrusion that are caused by cytoskeletal rearrangements, probably resulting from integrin outside-in signaling, could be observed, though. The limited impact on PS exposure as well as platelet spreading may result in part from the fact that FK633 in high doses has a partial agonistic effect for thromboxane A_2_ formation in thrombin stimulated platelets, which is a consequence of ligand-induced binding site expression on the β_3_ subunit of α_IIb_β_3_ [29]. In addition, FK633 is a specific α_IIb_β_3_ antagonist, and therefore there is a possibility that another integrin such as α_V_β_3_ [30] or α_5_β_1_ [31] could bind fibrin/fibrinogen as well. In spite of these facts, FK633 significantly reduced PS exposure on platelets, suggesting that outside-in signals in platelets generated by their binding to the rigid fibrin network are important for PS exposure. Similarly to FK633, we could clearly observe reduced kinetics of ANX binding to platelets by RGDS. In contrary, RGES, used as a control tetrapeptide, showed less significant inhibitory effect on both collagen-induced platelet aggregation and platelet PS exposure in CLSM studies. Our results are in good agreement with the previous reports showing that platelet procoagulant activity can be modulated by the α_IIb_β_3_ signaling [32], [33], [34]. Razmara and colleagues [35] found that α_IIb_β_3_ blockade attenuated inhibition of aminophospholipid translocase activity and limited scramblase activity in thrombin stimulated platelets. Moreover, Weiss and Lages [36] implied that α_IIb_β_3_ may play an important role in maintaining a sufficiently elevated [Ca^2+^]_i_ level. In support of our findings, van der Meijden and colleagues [37] demonstrated a constitutive role of α_IIb_β_3_ in [Ca^2+^]_i_ signaling and platelet PS exposure. In addition, they also showed a significant role of α_IIb_β_3_-dependent signaling via Syk kinase in TF-evoked platelet procoagulant activity in plasma. Taking all these results into consideration, α_IIb_β_3_ inhibitors appeared to attenuate the integrin outside-in signaling pathway, which involves [Ca^2+^]_i_ elevation and possibly Syk activation, resulting in the suppression of platelet PS exposure as a consequence of limited scramblase activity and reasonably maintained translocase activity.

In order to generate mechanical foci by cytoskeletal rearrangements in platelets, the binding and immobilization of platelets to a solid matrix seem to be required. To elucidate the importance of the fibrin network structure for bound platelets to expose PS, we employed GPRP, which interferes with fibrin polymerization and network formation. GPRP successfully inhibited fibrin polymerization even after thrombin treatment, but the platelets were still able to bind fibrin/fibrinogen, probably due to the platelets' activation by thrombin. Platelets also formed small aggregates, changed their shape and spread on the bottom of the dish. Since the presence of GPRP had a major inhibiting effect on platelet PS exposure in CLSM experiments (Figure 5B), even though thrombin's activity is not inhibited by GPRP, we presume that thrombin-evoked surface exposure of platelet anionic phospholipids requires mechanical foci generated by the surrounding fibrin network.

It has been reported that GPRP may inhibit fibrinogen binding to the α_IIb_β_3_ receptor [25], [26]. Representative images in Figure 4, however, show that the majority of platelets bound to fibrin/fibrinogen in CLSM experiments upon thrombin stimulation in the presence of GPRP, and this binding was entirely abolished by a combination of GPRP together with FK633. These results show that in our experimental model GPRP could have only a limited inhibitory effect on platelet binding to fibrin/fibrinogen and that it does not substantially impair integrin signaling. This again suggests that the binding of either soluble fibrin or fibrinogen to α_IIb_β_3_ is not sufficient for platelets to expose PS after thrombin stimulation. The binding of platelets to the fibrin scaffold seems to be important for thrombin activated platelets to expose PS on their cell surface.

A number of studies have demonstrated interdependence between the platelet cytoskeleton and the integrin α_IIb_β_3_. On the one hand, cytoskeletal reorganizations stabilize the interaction between integrin and its ligand, and on the other hand, ligand binding to the α_IIb_β_3_ initiates a series of intracellular alterations including reorganization of the cytoskeleton, as manifested by the formation of actin stress fibers, focal adhesion and clot retraction [38], [39], [40]. Our CLSM study revealed that platelets stimulated by thrombin and bound to the fibrin matrix retained the ability to generate contractile mechanical foci and to retract fibrin fibers (Video S1). Supplementation with Cyt-B dramatically reduced exposure of PS (Figure 5B), suggesting that cytoskeletal rearrangements and their results, such as α_IIb_β_3_ signaling and conformational changes or clot retraction, play an essential role in the exposure of negatively charged phospholipids in the platelet outer membrane leaflet.

Here we introduced a new approach for the *in vitro* evaluation of the generation of platelet procoagulant activity by measuring the kinetics of PS exposure in the platelet outer membrane leaflet. Our data confirm that PS exposure evoked by TF or thrombin is [Ca^2+^]_i_ dependent and precisely regulated by time- and space-dependent mechanisms. Integrin outside-in signals generated by platelet binding to a rigid fibrin network, and resulting in generation of mechanical foci through this binding, appear to be essential for modulation of anionic phospholipids' exposure. Such regulation of platelet PS exposure may play an important physiological role in the control of hemostasis.

## Supporting Information

Figure S1 **Aggregation profiles of non-labeled (open square) and R-6G-labeled (closed square) platelets.** (A) ADP-evoked (4.5 µM, n = 3) aggregation, and collagen-evoked aggregation (5 µg/ml, n = 3) (B) and (0.18 mg/ml, n = 3) (C). Data are shown as mean ± SD.
(TIF)

Figure S2 **RGDS (closed circle, 1.2 mM) inhibitory effect on platelet (A) ADP-evoked (4.5 µM, n = 3) and (B) collagen-evoked (0.18 mg/ml, n = 3) platelet aggregation in comparison with RGES (opened diamonds, 1.2 mM, n = 3) and control (closed square, n = 3).** (C) Kinetics of platelet PS exposure expressed as the percentage of ANX fluorescence-positive platelets in thrombin-treated (1 U/ml) samples in CLSM study. The effect of RGDS (open circle, 1.2 mM, n = 5) and RGES (open diamonds, 1.2 mM, n = 3) compared with control (close square, n = 7). Data are shown as mean ± SD.
(TIF)

Video S1 **Generation of apparent retractile foci by thrombin stimulated platelets bound to fibrin network.** Representative video shows typical interaction between platelets and fibrin network in CLSM studies. Fibrin mesh formation was followed by platelet shape changes accompanied by protrusion of pseudopods, which generated retractile foci to stretch surrounding fibrin fibers. Ultimately, blebbing and subsequent shedding of lipid-symmetric microvesicles from the cell surface were recognized.
(AVI)
