# Supplementary figures and images for: Binding of Thrombin-Activated Platelets to a Fibrin Scaffold through αIIbβ3 Evokes Phosphatidylserine Exposure on Their Cell Surface
Source: PLoS One. 2013 Feb 1;8(2):e55466. doi: 10.1371/journal.pone.0055466 (PMC3562181; doi:10.1371/journal.pone.0055466)

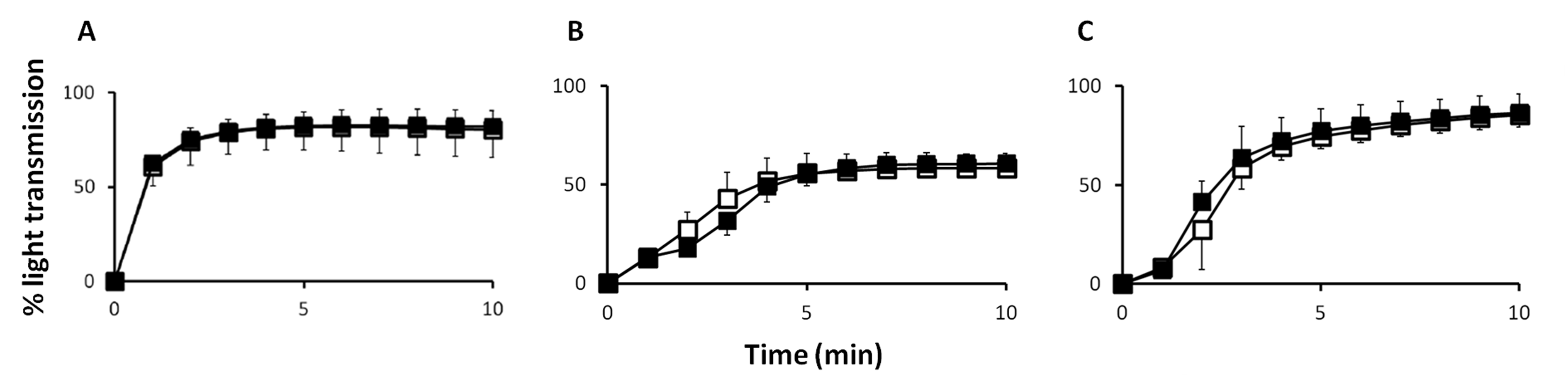

Supplement: Figure S1 — Aggregation profiles of non-labeled (open square) and R-6G-labeled (closed square) platelets. (A) ADP-evoked (4.5 µM, n = 3) aggregation, and collagen-evoked aggregation (5 µg/ml, n = 3) (B) and (0.18 mg/ml, n = 3) (C). Data are shown as mean ± SD. (TIF) [file pone.0055466.s001.tif]

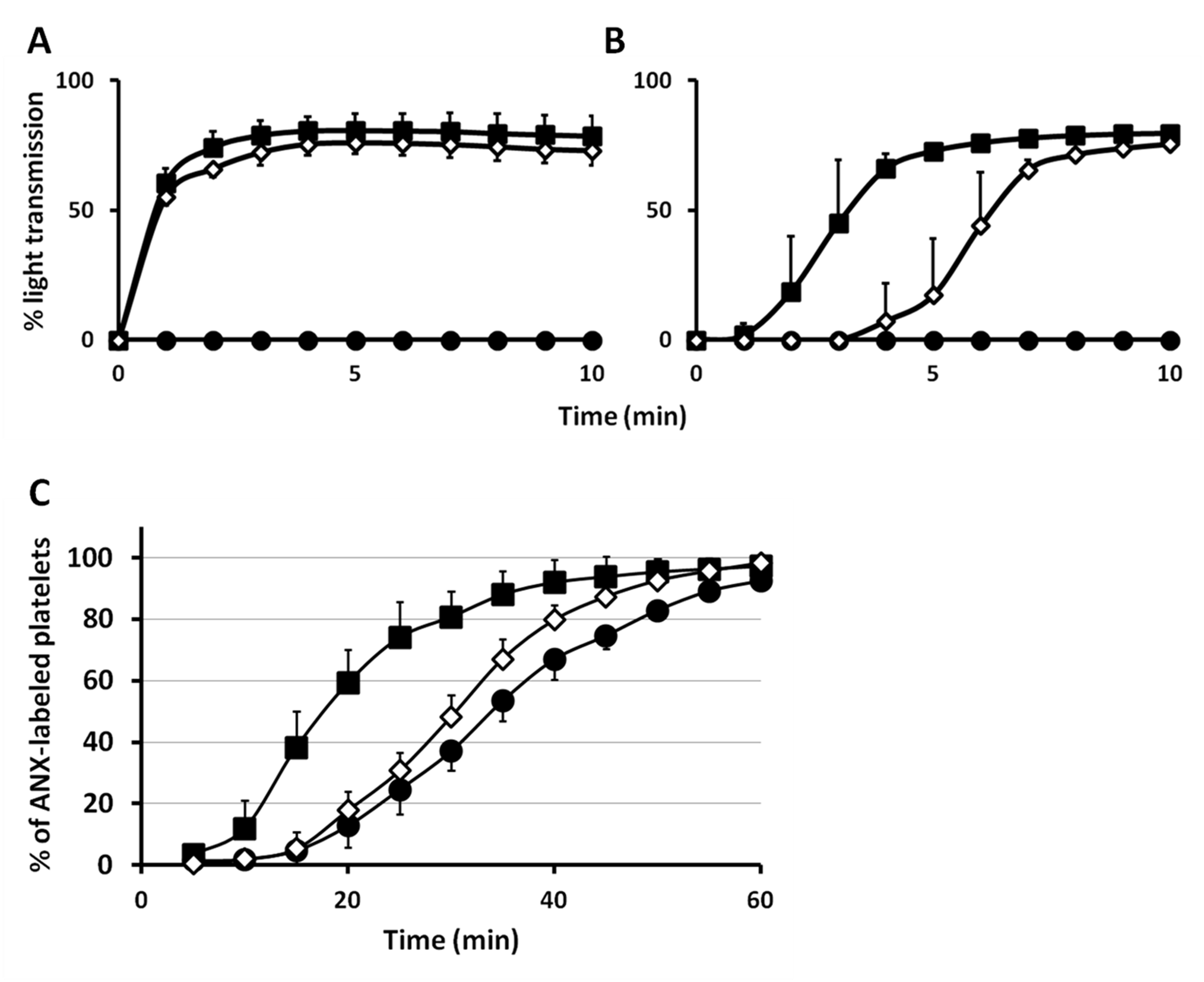

Supplement: Figure S2 — RGDS (closed circle, 1.2 mM) inhibitory effect on platelet (A) ADP-evoked (4.5 µM, n = 3) and (B) collagen-evoked (0.18 mg/ml, n = 3) platelet aggregation in comparison with RGES (opened diamonds, 1.2 mM, n = 3) and control (closed square, n = 3). (C) Kinetics of platelet PS exposure expressed as the percentage of ANX fluorescence-positive platelets in thrombin-treated (1 U/ml) samples in CLSM study. The effect of RGDS (open circle, 1.2 mM, n = 5) and RGES (open diamonds, 1.2 mM, n = 3) compared with control (close square, n = 7). Data are shown as mean ± SD. (TIF) [file pone.0055466.s002.tif]
